# Intense Activity of the Raphe Spinal Pathway Depresses Motor Activity via a Serotonin Dependent Mechanism

**DOI:** 10.3389/fncir.2017.00111

**Published:** 2018-01-09

**Authors:** Jean-François Perrier, Hanne B. Rasmussen, Lone K. Jørgensen, Rune W. Berg

**Affiliations:** ^1^Department of Neuroscience, Faculty of Health and Medical Sciences, University of Copenhagen, Copenhagen, Denmark; ^2^Department of Biomedical Science, Faculty of Health and Medical Sciences, University of Copenhagen, Copenhagen, Denmark

**Keywords:** serotonin, central fatigue, spinal cord, motor behavior, motor control, turtle, motoneuron, gain modulation

## Abstract

Motor fatigue occurring during prolonged physical activity has both peripheral and central origins. It was previously demonstrated that the excitability of motoneurons was decreased when a spillover of serotonin could activate extrasynaptic 5-HT_1A_ receptors at the axon initial segment (AIS) of motoneurons. Here we investigated the impact of massive synaptic release of serotonin on motor behavior in an integrated preparation of the adult turtle performing fictive scratching behaviors. We found that a prolonged electrical stimulation of the raphe spinal pathway induced a reversible inhibition of the motor behavior that lasted several tens of seconds. The effect disappeared when the spinal cord was perfused with an antagonist for 5-HT_1A_ receptors. By demonstrating a direct impact of serotonin on motor behavior, we suggest a central role of this monoamine behind central fatigue.

## Introduction

Prolonged physical activity results in the induction of motor fatigue. This temporary inability of muscles to operate maximally is caused by physically distinct mechanisms. In muscles, depletion of glycogenic reserve and failures of neurotransmitter release at neuromuscular junctions induce a weakening of contraction. Nevertheless, several lines of evidence obtained during the past century have demonstrated that fatigue also has a central origin. First, during the decline in force recorded in muscles during repeated voluntary contractions, it is possible to produce further muscular response by stimulating motor nerves directly (Reid, 1927). Second, during prolonged maximal voluntary contractions (MVCs), the exerted force declines steadily. Because it is possible to produce a motor twitch superimposed to the contraction by stimulating the nerve, it has been concluded that the central nervous system fails to recruit all the force that muscle could produce (Merton, 1954). Importantly, the amplitude of superimposed twitches observed during repeated stimulation gets larger during MVC, indicating that the level of central fatigue increases in the course of contraction (McKenzie et al., 1992; Gandevia et al., 1996; Gandevia, 2001). Third, in subjects maintaining a constant firing rate for a single motor unit for which they receive an audio feedback during a moderate isometric contraction, the force produced by the muscle increases. This shows that during prolonged activity, motoneurons require stronger excitatory inputs to maintain their firing rate (Johnson et al., 2004). In addition, it demonstrates that central fatigue does not require strong contractions in order to occur.

Evidence obtained in humans suggests that central fatigue occurs partly in the spinal cord. Indeed, the size of the muscle response evoked by stimulation of the corticospinal tracts at the cervicomedullary level decreases during MVC (Butler et al., 2003). This result indicates that prolonged efforts induce a decrease in the excitability of motoneurons. The mechanisms responsible for central fatigue remained hypothetical for many years. Among different conjectures, the idea that serotonin (5-HT) was involved in the induction of fatigue was supported by experiments performed on animals and humans. First, the injection of the serotonin precursor tryptophan in the blood of horses or directly in the brain of rats running on a treadmill accelerated the induction of exhaustion (Farris et al., 1998; Soares et al., 2003). Second, human subjects performing intense motor tasks reported higher levels of fatigue after intake of a selective serotonin reuptake inhibitor (Wilson and Maughan, 1992). Similar results were obtained after oral ingestion of a 5-HT_1A_ receptor agonist (Marvin et al., 1997). Third, the excitability of motoneurons estimated by counting the occurrence of F-waves induced by peripheral stimulation, declined during central fatigue. However, the effect disappeared when subjects ingested an agonist for 5-HT_1A_ receptors (D’Amico et al., 2017).

**Figure 1:**
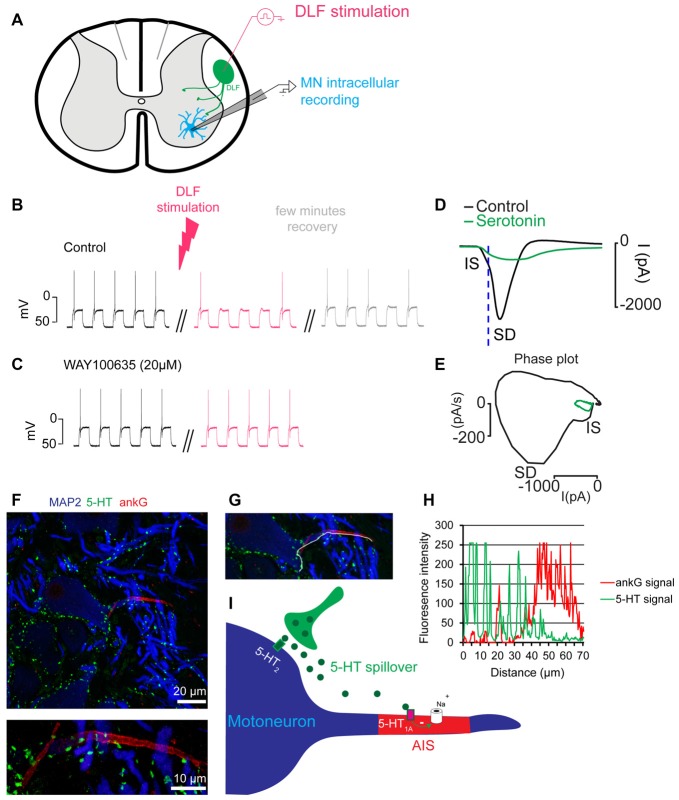
Prolonged synaptic release of serotonin decreases the excitability of motoneurons. **(A)** Scheme of a slice preparation from the spinal cord of the adult turtle. A motoneuron is recorded intracellularly. The raphe spinal pathway is activated by electrical stimulation of the dorsolateral funiculus (DLF). **(B)** Membrane potential of a motoneuron in response to depolarizing current pulses. In control condition, each pulse generates one action potential. After prolonged stimulation of the DLF (4 min, 8 Hz) the excitability is decreased (red upper trace). The effect is reversible after few minutes (gray). **(C)** In the presence of the 5-HT_1A_ receptor antagonist WAY100635 (20 μM), stimulation of the DLF does not decrease the excitability of the motoneuron (lower traces). Results in **(A–C)** are adapted from Cotel et al. (2013). **(D)** Unclamped action potential from a motoneuron recorded in voltage clamp mode. Serotonin applied near the initial segment (IS) inhibited the inward current (green). **(E)** Phase plot representing the first derivative of the current as a function of the membrane current. This representation allows a clear distinction of the spike generated at the IS from the spike back-propagating in the somato-dendritic compartment (SD). After serotonin application, the IS component is inhibited and the SD component disappears in an all or none manner. Results in **(D,E)** are adapted from Petersen et al. (2016). **(F)** Turtle spinal cord section triple-labeled for 5-HT (green), ankyrin G (ankG, red, marker of the axon IS (AIS)) and microtubule-associated protein-2 (MAP2, blue, marker of soma and dendrites). The image is recorded from the ventral horn of the spinal cord and centers the soma of a motoneuron. The upper image represents a maximum projection of five consecutive confocal scans covering 4 μm in the vertical plane to include the entire AIS (located in different focal planes). The lower image represents a maximum projection of confocal scans of the same AIS, recorded at higher optical zoom. **(G)** Image illustrating the line (white) plotted to obtain the intensity profile in **(H)**. **(H)** Plot of the fluorescence intensity profile of the 5-HT and ankG labeling along the white line shown in **(G)**. The line runs along the edge of the somatodendritic compartment and continues in the AIS. Note the number of high intensity 5-HT profiles along the somatodendritic compartment that abruptly drop when entering the AIS. Notice that the high intensity ankG peak at 20 μm belongs to that of a crossing AIS from another cell. **(I)** Scheme of the cellular mechanism responsible for central fatigue.

The demonstration of the serotonin hypothesis was made by studies performed *in vitro* on preparations from a non-mammalian vertebrate. In slices and integrated preparations from the spinal cord of adult turtles, a brief stimulation of the raphe spinal pathway, which releases serotonin on motoneurons, increased the excitability of motoneurons by activating 5-HT_2_ receptors (Perrier and Delgado-Lezama, 2005; Perrier and Cotel, 2008, 2015; Cotel et al., 2013; Perrier, 2013, 2016). These results were in agreement with older observations obtained *in vivo* in the cat (Hounsgaard et al., 1988). In contrast, a prolonged stimulation of the raphe spinal pathway induced a decrease in the excitability of motoneurons (Figures 1A–C; Cotel et al., 2013). This effect was mediated by the activation of 5-HT_1A_ receptors located at the axon initial segment (AIS) of motoneurons (Cotel et al., 2013). The axonal serotonergic receptors inhibited the sodium ion channels responsible for the genesis of action potentials (Figures 1D,E; Cotel et al., 2013; Petersen et al., 2015). Somatodendritic compartments of spinal motoneurons are densely innervated by serotonergic synaptic boutons (Figures 1F,G; Kiehn et al., 1992; Alvarez et al., 1998; Cotel et al., 2013). In contrast, the AIS is devoid of serotonergic innervation (Figure 1H; Cotel et al., 2013; Montague et al., 2013). To understand how serotonin can affect a compartment without synaptic boutons it is important to realize that the activity of raphe spinal neurons is directly correlated to the level of motor activity (Veasey et al., 1995) and that prolonged stimulations of the raphe spinal pathway trigger a spillover of serotonin (Cotel et al., 2013). For these reasons, it was concluded that during moderate motor tasks, serotonin is released on motoneurons where it activates intrasynaptic receptors that promote the excitability of motoneurons (Perrier and Hounsgaard, 2003; Cotel et al., 2013). When the level of motor activity increases, a spillover allows serotonin to reach 5-HT_1A_ receptors at the AIS and to induce central fatigue (Figure 1I; Cotel et al., 2013; Perrier and Cotel, 2015; Perrier, 2016; Petersen et al., 2016).

The decrease of motoneuron excitability induced by massive synaptic release of serotonin is well documented. However, its specific impact on motor behavior remains to be determined. Indeed, serotonin modulates the excitability of several types of interneurons located in the ventral horn of the spinal cord (Perrier and Cotel, 2015) and its overall effect on motor control is rather heterogeneous. Different studies have reported that serotonin promotes locomotion (Cazalets et al., 1992; Brustein et al., 2003), while others found that it exerts an inhibitory effect (Beato and Nistri, 1998; McLean and Sillar, 2004; Dunbar et al., 2010). It is therefore difficult to predict the overall effect of a massive synaptic release of serotonin on the output produced by the whole motor neuronal network.

Here we investigated this question in an integrated preparation of the adult turtle that can generate reproducible fictive scratching (Alaburda and Hounsgaard, 2003; Vestergaard and Berg, 2015). We show that a prolonged stimulation of the raphe spinal pathway inhibits movement production via activation of 5-HT_1A_ receptors.

## Materials and Methods

### Integrated Preparation of Spinal Cord and Carapace

Red-eared turtles (*Trachemys scripta elegans*) were placed on crushed ice for 2 h to ensure drowsiness and reduce stress and pain by hypothermia. The head could be protracted using minimal force. Animals were killed by decapitation and brain functions were terminated by crushing the head. The heart was perfused with Ringers solution containing (mM): 120 NaCl; 5 KCl; 15 NaHCO_3_; 2 MgCl_2_; 3 CaCl_2_; and 20 glucose, saturated with 98% O_2_ and 2% CO_2_ to obtain pH 7.6. The carapace containing the D4-D10 spinal cord segments was isolated by transverse cuts (Petersen and Berg, 2017). This study was carried out in accordance with the recommendations of Department of Experimental Medicine of the University of Copenhagen. The protocol was approved by the Danish veterinary and food administration with the ministry of environment and food.

### Recordings

**Figure 2:**
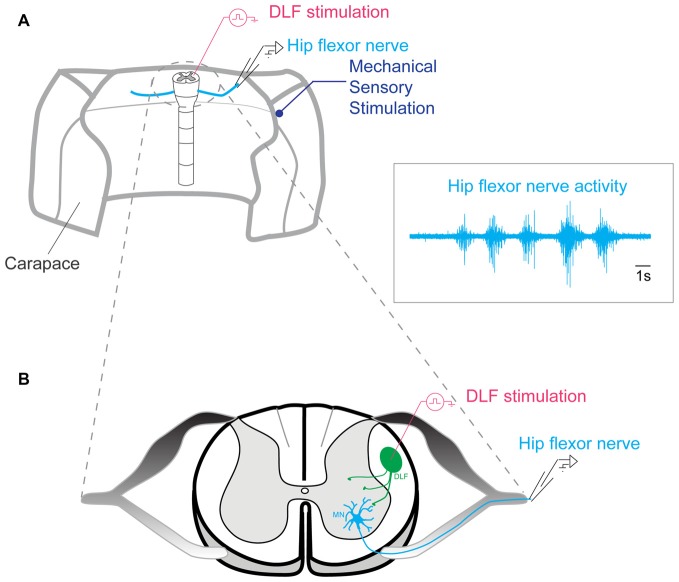
Preparation used for generating scratch behavior.** (A)** Lumbar segments of the spinal cord left intact in the carapace of an adult turtle. Scratch episodes evoked by mechanical stimulation of the skin were monitored by recording a hip flexor nerve activity with a suction electrode. DLF stimulated by a bipolar electrode. Insert: example of scratch reflex recorded in the hip flexor nerve. **(B)** Representation of the spinal cord section seen from top.

The spinal column was continuously perfused with Ringers solution. The activity of a hip flexor nerve was recorded with a suction electrode and amplified by a differential amplifier Iso-DAM8 (WPI). It was sampled at 20 kHz with a 12-bit analog-to-digital converter (Digidata 1200, Axon Instruments, Union City, CA, USA), displayed by means of Axoscope and Clampex software (Axon Instruments, Union City, CA, USA), and stored on a hard disk. The bandwidth was 100–1 kHz (Figure 2A).

### Network Activation

Scratch reflexes were induced via mechanical cutaneous touch on turtle skin and carapace with a glass rod mounted to the membrane of a loudspeaker driven by sinusoidal current. This procedure induced reproducible series of 4–6 burst of activity (inset in Figure 2A) corresponding to scratch reflexes (Alaburda and Hounsgaard, 2003; Alaburda et al., 2005; Petersen et al., 2014; Petersen and Berg, 2016).

### Extracellular Stimulation of the Dorsolateral Funiculus

Activation of the dorsolateral funiculus (DLF), which contains the raphe spinal pathway, was accomplished with electrical stimulation by means of bipolar concentric micro-electrodes (World Precision Instruments, Inc., Sarasota, FL, USA) in segment D10 on the cut surface in the appropriate location (Figure 2B). A train of 100 μs pulses was given at 5–8 Hz during 1 min. The current was adjusted in individual experiment from 0.3–2 mA such that no excessive electroneurogram (ENG) activity was induced while a small non-rhythmic DC component could be observed.

### Drug Delivery

The 5-HT_1A_ receptor antagonist WAY 100635 (10 μM) was delivered via perfusion through the spinal column.

### Cloning of HA-Tagged Human 5-HT_**1A**_

An hemagglutinin (HA) tag was inserted into the N-terminus of pXOOM-h5-HT_1A_ (NM_000524). pXOOM-5-HT_1A_ has been described previously (Grunnet et al., 2004). The HA-tag was introduced by PCR amplification using the primers atagcggccgctcactggcggcagaacttacac and atagaattcccaccatgtacccatacgatgttccagattacgctggtagtggtagtggtagtgatgtgctcagccctggtcag and inserted into pXOOM using ECoRI and NotI. A linker sequence of six amino acids (GSGSGS) between the HA-tag and the coding sequence of h5-HT_1A_ was included. The plasmid was verified by complete DNA sequencing of the cDNA insert (Macrogen Inc., Seoul, South Korea).

### Primary Antibodies

The primary antibodies utilized were: rat anti-serotonin antibody (1:100 dilution, clone YC5/45, Merck), rabbit anti-ankyrin-G (anti-ankG; 1:300 dilution, H-215, Santa Cruz Biotechnology), mouse anti-microtubule-associated protein-2 (anti-MAP-2; 1:250 dilution, A-4, Santa Cruz Biotechnology), goat anti-choline acetyltransferase (anti-ChAT; 1:100 dilution. AB144P, Merck Life Science A/S), rabbit anti-5-HT_1A_ (1:100, H-119, Santa Cruz Biotechnology), rabbit anti-5-HT_1A_ (1:50, ASR-021, Alomone Labs), rabbit anti-5-HT_1A_ (1:100, ADI905-741-100, Enzo Life Sciences), rabbit 5-HT_1A_ (1:100, NB100-92418, Novus Biologicals), and mouse anti-HA epitope (1:500 dilution, clone 16B12, Biolegend). Rabbit 5HT_1A_ S1A-170 serum (1:1000–1:2500 dilutions) was a generous gift from Professor Efrain C. Azmitia, New York University, New York, NY, USA.

### HEK Cell Cultures, Transfections and Immunocytochemistry

HEK293 cells were cultured in DMEM (in-house, Faculty of Health and Medical Sciences) supplemented with 100 U/mL penicillin, 100 mg/mL streptomycin (ThermoFischer Scientific) and 10% fetal calf serum (FCS, Sigma-Aldrich) at 37°C in 5% CO_2_. Cells were transfected with pXOOM-h5-HT_1A_ using Lipofectamine and Plus Reagent (ThermoFischer Scientific) according to the manufacturer’s instructions. Cells were fixed 24–48 h after transfection in 4% paraformaldehyde in PBS for 10 min. They were blocked and permeabilized for 30 min in PBS containing 0.1% Triton X-100 and 0.2% fish skin gelatin and then incubated for 1 h with primary antibodies diluted in the same buffer. AlexaFluor^®^-conjugated secondary antibodies (ThermoFisher Scientific) were applied for 45 min at room temperature and 4′,6-diamidino-2-phenylindole (DAPI, ThermoFisher Scientific) was used to stain nuclei. Coverslips were mounted on glass slides with Prolong Gold Antifade Reagent (ThermoFisher Scientific).

### 5HT_**1A**_ Knockout Animals

Brains from 5-HT_1A_ knockout and control 129/Sv mice were generously provided by Dr. Mark A. Geyer, Center for Neurobiology and Behavior, Columbia University, New York, NY, USA. These mice have been described previously (Dirks et al., 2001). The fixed brains were cryoprotected for 3 days in 30% sucrose in PBS, frozen in dry ice and cut in 40 μm frontal sections on a cryostat. Free-floating sections were stored at 4°C in PBS + 0.1% sodium-azide prior to use. Blocking was performed by a 30 min incubation in blocking buffer (4% low-fat milk powder, 0.3% Triton X-100 in PBS). The sections were subsequently incubated with primary antibodies overnight at 4°C. AlexaFluor^®^-conjugated secondary antibodies were applied for 2 h at room temperature and DAPI included to stain nuclei. Sections were mounted in Prolong Gold Antifade Reagent (ThermoFisher Scientific).

### Immunohistochemistry

Turtle spinal cords were fixed at 4°C overnight in 4% paraformaldehyde in PBS. After washing in PBS, cryoprotection was carried out by two successive overnight incubations at 4°C in PBS supplemented with 20% (w/v) and then 30% (w/v) sucrose. Ten to fifteen micrometer cryostat sections were cut. Prior to staining, sections were subjected to antigen retrieval by boiling in 10 mM citric acid pH 6.0 for a total of 5 min (3 + 2 min with a 1 min break in-between). Unspecific binding was blocked for 30 min in PBS containing 0.1% Triton X-100 and 0.2% fish skin gelatin. Primary antibodies were diluted in the same buffer and incubated overnight at 4°C. AlexaFluor^®^-conjugated secondary antibodies (ThermoFisher Scientific) were applied for 1 h at room temperature to detect bound primary antibody. Sections were mounted in Prolong Diamond (ThermoFisher Scientific).

### Confocal Microscopy

Confocal images were captured using a Zeiss LSM 710 confocal microscope with a ×63 oil immersion objective (NA 1.4). Pinhole size was set to 1, the pixel format was 1024 × 1024. Line averaging was employed to reduce noise. Maximum projections and fluorescence intensity profiles were created using ZEN 2012 (Black edition).

### Statistical Analysis

**Figure 3:**
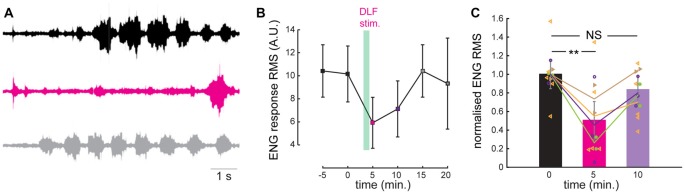
A prolonged stimulation of the DLF inhibits the scratch reflex.** (A)** Electroneurogram (ENG) of a hip flexor nerve during scratch reflex evoked by mechanical stimulation of the skin. Upper trace (black): control reflex. Middle trace (purple): scratch reflex evoked shortly after a 1 min stimulation of the DLF at 5 Hz. Lower trace (gray): recovery recorded 5 min after the beginning of DLF stimulation. **(B)** Time course of the effect induced by DLF stimulation. Scratch amplitude quantified by calculating the root-mean-square (RMS) of the ENG during full scratch episodes. DLF stimulation produced a strong and reversible inhibition of the scratch reflex (individual points are the average of 2 or 3 trials). **(C)** Comparison of the normalized ENG amplitude for all the preparations used for the study (*n* = 4) in control conditions, after DLF stimulation and after recovery. Significant inhibition induced by DLF. **p < 0.01.

**Figure 4:**
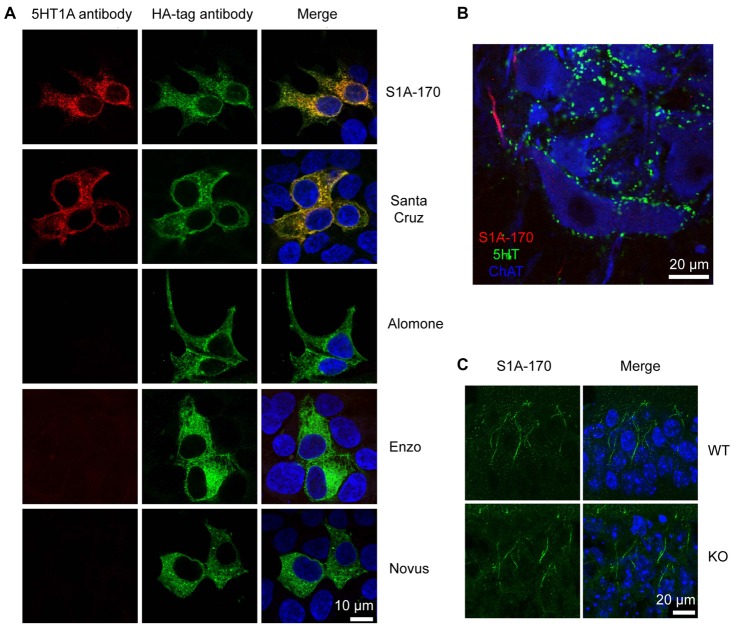
Unspecificity of available 5HT_1A_ antibodies.** (A)** HEK293 cells transfected with HA-tagged h5-HT_1A_ and stained with anti-HA antibodies in combination with five different anti-5-HT_1A_ antibodies. Anti-5-HT_1A_ staining (red) obtained using the five antibodies is depicted in the left column, while anti-HA (green) is shown in the middle column. Only two antibodies were able to recognize HA-h5-HT_1A_ expressing HEK293 cells, S1A-170 and H-119 from Santa Cruz Biotechnology. The remainder of the antibodies revealed either no staining or unspecific staining. Staining of the nuclei with 4′,6-diamidino-2-phenylindole (DAPI) is included in blue in the merged images to the right. **(B)** Turtle spinal cord section triple-labeled with S1A-170 (red), 5-HT (green) and choline acetyltransferase (ChAT, blue, marker of motorneurons). S1A-170 stained the proximal region on a motoneuron neurite consistent with the previously reported AIS staining with this antibody. **(C)** Mouse hippocampal CA1 wild-type and knockout tissue sections were stained with S1A-170 (green). As shown, stainings consistent with the localization of the AIS was observed in pyramidal cells of wild-type animals. The staining was, however, not eliminated in 5-HT_1A_ knockout animals demonstrating that this staining is not specific for 5-HT_1A_. The right column represents overlay images of S1A-170 immunoreactivity and DAPI signal (blue).

Data were analyzed by means of Origin 7.5 (Origin Lab, USA) and GraphPad Prism version 6.0 (GraphPad, San Diego, CA, USA). The motor nerve output, the ENG, was measured as a surrogate for the muscular force (Vestergaard and Berg, 2015). The ENG signal was digitally filtered in MATLAB using a nine pole Butterworth bandpass filter. The root-mean-square (RMS) of the filtered ENG signal formed the basis of the analysis. This background signal was subtracted to quantify and distinguish the increase in nerve activity from the noisy background. For parts of the analysis (Figures 3, 4) the signal was normalized to the control situation in order to compare a change in the response across animals.

The normality of distribution of each set of data was tested and in cases when a Gaussian distribution could not be approximated, a non-parametric test was used. For parametric data, one-way analysis of variance (ANOVA) were followed by Tukey *post hoc* tests. When normal distribution could not be approximated, we used a Kruskal-Wallis followed by Dunn’s multiple comparison test. Data are represented as mean ± standard deviation (SD) of the mean.

## Results

We induced fictive scratching in an integrated preparation consisting of the lumbar spinal cord left intact in the backbone and the carapace of the adult turtle (Alaburda and Hounsgaard, 2003; see “Materials and Methods” section). A brief mechanical stimulation of the skin pocket near the hip produced a rhythmic activity corresponding to a scratch motor behavior. We monitored the scratch episodes by recording a hip flexor nerve on the ipsilateral side (Figure 3A) and quantified the response by calculating the root mean square of the electroneugram. Scratch reflexes evoked every 5 min were highly reproducible as shown in previous studies (Vestergaard and Berg, 2015). We tested the effect of a prolonged synaptic release of serotonin by stimulating the DLF at 5–8 Hz (pulses of 0.3–2 mA) during 1 min. These frequencies are well within the range of neuronal firing recorded in raphe nuclei recorded *in vivo* during motor activities (Veasey et al., 1995). For the preparation illustrated, as for all the preparations tested (*n* = 4), we found that 1 min after ending DLF stimulation, the amplitude of evoked scratch reflexes was strongly inhibited (Figures 3A–C; mean inhibition to 50 ± 40% of control value; *n* = 12 trials from four different preparation; *p* = 0.004; Kruskal-Wallis followed by Dunn’s multiple comparison test). Five minutes later, the motor behavior was still smaller than under control conditions, however the difference was not significant when compared to control conditions (Figures 3A–C; inhibition to 79 ± 23% of control value; *n* = 12 trials from four different preparation; *p* > 0.05; Kruskal-Wallis followed by Dunn’s multiple comparison test).

It has been shown that in case of serotonin spillover, the activation of 5-HT_1A_ receptors located at the initial segment (IS) of motoneurons decreases the excitability by inhibiting the Na^+^ current responsible for action potential genesis (Cotel et al., 2013).

We tested for the presence of these receptors by means of immunohistochemical staining performed with antibodies directed against 5-HT_1A_ receptors. We tested five different antibodies directed against different epitopes (from Alomone labs, Santa Cruz Biotechnology, Enzo Life Sciences, Novus Biologicals and in addition, the antibody S1A-170 kindly provided by Dr. Efrain C. Azmitia, New York University, New York, NY, USA).

We first tested whether the antibodies were able to recognize their target in HEK cells over-expressing 5-HT_1A_ receptors. The receptors were tagged with an HA epitope in the N-terminus. Only the S1A-170 (Azmitia) and the H-119 (Santa Cruz) antibodies showed positive immunoreaction overlapping with the HA immunoreactivity (Figure 4A). The remaining three antibodies did not show any specific immunoreaction. We then tested the S1A-170 and the H-119 antibodies on fixed slices from the spinal cord of the turtle. We obtained a staining only with the S1A-170 (Figure 4B), which labeled the proximal region of one motoneuron neurite. This result is consistent with a labeling of the AIS, as previously observed for primate motoneurons when using this antibody (Kheck et al., 1995). We next tested the specificity of the antibody by comparing the labeling obtained in CA1 regions from the hippocampus of wild-type and 5-HT_1A_ knockout animals. Stainings consistent with the localization of the AIS were observed in pyramidal cells of wild-type animals (Figure 4C). The staining was, however, not eliminated in 5HT_1A_ knockout animals (Figure 4C). This result demonstrates that the AIS staining is not specific for receptor. Overall, these negative observations are important because they demonstrate that none of the 5 tested antibodies provide an endogenous staining specific for 5-HT_1A_ receptors.

**Figure 5:**
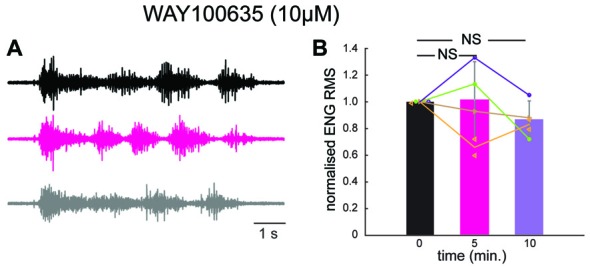
In the presence of a 5-HT_1A_ receptor antagonist, stimulation of the DLF does not inhibit the scratch reflex.** (A)** ENG of a hip flexor nerve during scratch reflex evoked by mechanical stimulation of the skin. Upper trace (black): control reflex. Middle trace (purple): scratch reflex evoked shortly after a 1 min stimulation of the DLF at 5 Hz. Lower trace (gray): recovery recorded 5 min after 5 min after the beginning of DLF stimulation. All recordings were performed in the presence of WAY 100635 (10 μM) perfused through the spinal column. **(B)** Comparison of the normalized ENG amplitude for all the preparations used for the study (*n* = 4) in control conditions, after DLF stimulation and after recovery. No significant inhibition induced by DLF.

We therefore decided to test if 5-HT_1A_ receptors were responsible for the inhibition induced by prolonged DLF stimulation by means of pharmacology. We perfused the preparation with a selective antagonist for 5-HT_1A_ receptors (WAY 100635; 10 μM). After addition of the compound, the four preparations tested were still able to generate scratch reflexes (Figure 5A). However, a prolonged stimulation of the DLF did not affect the amplitude of bursts anymore (Figures 5A,B; mean effect to 94 ± 30% of control value; *n* = 5 trials from four different preparation; *p* = 0.44; Kruskal-Wallis followed by Dunn’s multiple comparison test). This observation confirms the fact that the inhibitory effect induced by DLF stimulation is mediated by serotonin. In addition, it is compatible with the finding that the activation of 5-HT_1A_ receptors on motoneurons is essential for triggering central fatigue.

## Discussion

This study aimed at testing how prolonged activity of the raphe spinal pathway impacted motor behavior. Our results show that the release of serotonin produces a significant reduction of motor output, which is mediated by the activation of 5-HT_1A_ receptors.

### Technical Considerations

One could argue that the decrease in scratch amplitude is caused by the repetition of the motor behavior and not by the release of 5-HT induced by DLF stimulation. Two arguments convinced us that it is not the case. First, in agreement with previous studies made on the same preparation, we found that motor patterns evoked every 5 min evoked reproducible behaviors (Vestergaard and Berg, 2015). Second, by selectively blocking 5-HT_1A_ receptors, we prevented the induction of fatigue.

In addition to serotonergic fibers, DLF stimulation also induces the release of glutamate and acetylcholine (Delgado-Lezama et al., 1997). These neurotransmitters promote the excitability of motoneurons by activating metabotropic receptors that facilitate a persistent Ca^2+^ current (Delgado-Lezama et al., 1997; Alaburda et al., 2002; Perrier et al., 2002). It is therefore unlikely that they contribute to the decrease of motoneuron excitability.

Due to the lack of commercially available specific antibody directed against 5-HT_1A_ receptors, it is difficult to determine where the receptors are expressed in the spinal cord. It is though unlikely that the expression is restricted to motoneurons. Indeed, *in situ* hybridization studies suggest the presence of mRNA encoding for the receptor in some interneurons (Zhang et al., 1997; Talley and Bayliss, 2000). These receptors were also blocked when WAY 100635 was applied to the preparation and we cannot exclude that this contributed to the blockade of DLF induced fatigue. However, in a previous study we showed that the decrease in motoneuron excitability induced by prolonged DLF stimulation could be induced after blocking fast synaptic transmission in the spinal cord (Cotel et al., 2013). It is therefore likely that the effect of DLF on motoneurons is monosynaptic and that interneuronal contribution, if any, is minor.

### Heterogeneity of the Effects Induced by Serotonin

The net effects induced by serotonin applied on the spinal cord are heterogeneous and vary from facilitation of rhythmic activity to strong inhibition of locomotion (Cazalets et al., 1992; Beato and Nistri, 1998; Brustein et al., 2003; Dunbar et al., 2010). For this reason, we could not predict the overall effect of a massive synaptic release of serotonin on motor behavior before performing our experiments.

Serotonin acts by binding to different subtypes of receptors coupled to various intracellular pathways. Importantly, the receptors are not homogeneously expressed in neuronal compartments. Some of them have been detected in synapses while others have been found outside synaptic compartments (Perrier and Cotel, 2015). It is believed that moderate release primarily activates intrasynaptic receptors and that extrasynaptic receptors are only activated during massive release if a spillover occurs. The differential expression of receptors explains why the effects produced by endogenous and exogenous serotonin are different (Dunbar et al., 2010).

On motoneurons, extrasynaptic 5-HT_1A_ receptors are expressed at the AIS (Cotel et al., 2013). This location is particularly strategic as it is where action potentials of the final common output of the central nervous system are generated. The inhibition of sodium channels responsible for nerve impulse genesis produced by the activation of 5-HT_1A_ receptors is therefore likely to overcome any other modulatory effect produced by serotonin at other locations of the spinal cord.

Central fatigue has a spinal (Butler et al., 2003) and supraspinal components (Gandevia et al., 1996). Since serotonergic neurons project to most regions of the central nervous system, it is plausible that 5-HT alters the gain of different types of neurons. Marvin et al. (1997) found that the fatigue induced by buspirone was concomitant with an increase in serum prolactin. This suggests that the hypothalamus was activated by the 5-HT_1A_ receptor agonist. However, a link of causality between prolactin and fatigue was not established.

### Functional Implications

*In vivo* recordings of units from raphe nuclei in freely moving cats have shown that the firing rate is tightly correlated to the speed of the animal (Veasey et al., 1995). This suggests that the amount of serotonin released in the spinal cord increases in parallel with motor activity. Synaptic release of serotonin induced by brief (<1 s) stimulation of raphe spinal fibers consistently induces an increase in motoneuron excitability (Delgado-Lezama et al., 1997; Perrier and Hounsgaard, 2003; Perrier and Delgado-Lezama, 2005; Cotel et al., 2013). In contrast, prolonged stimulation of the raphe spinal pathway produces a decrease in excitability of motoneurons caused by the inhibition of the Na^+^ current responsible for action potential initiation in motoneurons (Cotel et al., 2013). Here we documented that this phenomenon is concomitant with a strong inhibition of motor behavior. From a functional point of view, it is plausible that weakening muscle contraction serves as a safety mechanism that prevents damages of the body during intense physical activities (Gandevia, 2001).

In addition, experiments performed on humans have shown that central fatigue also occurs during prolonged contractions of weak intensities. Subjects maintaining a constant firing rate for a single motor unit for which they received an audio feedback had to increase the motor drive to the muscle in order to achieve the task (Johnson et al., 2004). Based on this finding, it has been suggested that central fatigue is an important component of the rotation of motor units occurring during prolonged contractions (Bawa et al., 2014). Can the spillover of serotonin to the AIS of motoneurons be responsible for the rotation of motor units? This would imply that serotonin modulates first and foremost the AIS of active motoneurons. If the afferent pathways responsible for the activation of motoneurons had an axon collateral projecting on serotonergic fiber terminals, then the release of serotonin on active motoneurons would be promoted. In that case a central fatigue mechanism involving serotonin spillover could selectively inhibit the most active motoneurons and explain the rotation of motor units. This possibility, which remains to be demonstrated will be tested in future experiments.

## Author Contributions

RWB, HBR, LKJ and J-FP performed the experiments and wrote the manuscript. All authors approved the manuscript.

## Conflict of Interest Statement

The authors declare that the research was conducted in the absence of any commercial or financial relationships that could be construed as a potential conflict of interest.
